# Comparison of Complications in Early and Late Cranioplasty Following Decompressive Craniectomy Due to Traumatic Brain Injury: Systematic Review and Meta-Analysis

**DOI:** 10.3390/jcm14124176

**Published:** 2025-06-12

**Authors:** Oskar Gerald Chasles, Klaudia Kokot, Justyna Fercho, Mariusz Siemiński, Tomasz Szmuda

**Affiliations:** 1 Scientific Circle of Neurotraumatology, Department of Emergency Medicine, Medical University of Gdańsk, 80-210 Gdańsk, Poland; klaudia.kokot@gumed.edu.pl; 2 Department of Emergency Medicine, Medical University of Gdańsk, University Clinical Centre in Gdańsk, 80-952 Gdańsk, Poland; sieminski@gumed.edu.pl; 3 Neurosurgery Department, Swissmed Luxmed Hospital in Gdańsk, 80-215 Gdańsk, Poland; tszmuda@gumed.edu.pl; 4 Neurosurgery Department, 10th Military Clinical Hospital with PolyClinic SPZOZ in Bydgoszcz, 85-681 Bydgoszcz, Poland; 5 Neurosurgery Department, Medical University of Gdańsk, University Clinical Centre in Gdańsk, 80-952 Gdańsk, Poland

**Keywords:** cranioplasty, traumatic brain injury, decompressive craniectomy, complications

## Abstract

**Objectives:** This study investigates the relationship between the time elapsed from decompressive craniectomy to cranioplasty and surgical complications in patients after traumatic brain injury. **Methods:** PubMed, Scopus, and Web of Science were systematically searched for references using the PRISMA guidelines. The data were subjected to the first phase of screening, which required the studies to be published between 1990 and 2024, be written in English, and include patients who underwent cranioplasty following decompressive craniectomy due to traumatic brain injury. The second phase of screening assessed whether the studies included at least 10 patients and compared their outcomes based on the time between decompressive craniectomy and cranioplasty. A subgroup analysis was performed for ultra-early cranioplasty patients. **Results:** This meta-analysis included fifteen studies involving patients who underwent early (*n* = 666) and late cranioplasty (n = 1214) after decompressive craniectomy. All studies had a retrospective observational design. There was no statistically significant difference in the odds of complications between the groups, although late cranioplasty had slightly elevated odds of developing hydrocephalus (OR 1.66, 95% CI 0.55–4.99, *p* = 0.36). Interesting results stemmed from a subgroup analysis of the ultra-early cohort; they included favoring the ultra-early group in the odds of overall complications (OR 0.46, 95% CI 0.08–2.56, *p* = 0.38) and hygroma (OR 0.45, 95% CI 0.15–1.37, *p* = 0.16). Later cranioplasty had better outcomes in the category of seizure (OR 1.56, 95% CI 0.75–3.28, *p* = 0.24). **Conclusions:** Cranioplasty within 90 days, considered early, had no statistically significant differences in complication rates compared to late cranioplasty.

## 1. Introduction

Decompressive craniectomy (DC) is one of the treatment methods used in patients presenting with traumatic brain injury (TBI). It involves reducing intracranial pressure by excising an appropriately sized fragment of the patient’s skull. The initial surgery is then followed by performing a cranioplasty (CP) to replenish the skull defect, thus restoring cerebral protection, improving neurological status [1], and addressing the cosmetic aspect. The intervals between DC and CP vary greatly [2]. TBI is becoming a global health priority as yearly incidence is rising [3]. Therefore, determining an optimal time for CP may improve the surgical outcomes of future patients.

Previous meta-analyses presented limitations, including insufficient subjects [4] or not being directly focused on TBI cases [5]. The small number of studies presenting adequate patient data restricted statistical outcomes. Studies encompassing mixed indications for DC could provide general data, which would not always be applicable in TBI cases, due to their specificity. The debate about the timing of CP and its effects on patients’ outcomes remains open. Recent studies have introduced the term “ultra-early cranioplasty”, yet no universally accepted criteria exist.

As new cohort studies have been published in recent years, this paper aims to present a more conclusive overview of the rate and type of surgical complications relative to the timing of CP in trauma patients, and further analyze the link between ultra-early cranioplasty and complications, through systematic review and meta-analysis.

## 2. Methods

### 2.1. Search Strategy and Protocol

We systematically screened PubMed, Scopus, and Web of Science databases following the Preferred Reporting Items for Systematic Reviews and Meta-Analyses (PRISMA) guidelines. The search, performed separately by two authors (O.G.C. and K.K.), focused on studies containing the following keywords in their title or abstract: cranioplasty and traumatic brain injury, including abbreviations. Conflicts at this stage were resolved via arbitration by two more experienced authors (J.F. and T.S.). Possible references published between 1 January 1990 and 5 January 2024 were extracted for screening. The protocol for this systematic review was registered on PROSPERO (CRD42024621539 (https://www.crd.york.ac.uk/PROSPERO/view/CRD42024621539, accessed on 29 May 2025).

### 2.2. Selection Criteria

The first stage of screening, which involved assessing titles and abstracts of studies, necessitated the studies to be written in the English language and report postoperative complications in patients who underwent DC and CP due to TBI. Studies that passed the first screening stage were subjected to the second stage, which included full-text screening. The studies were required to include 10 or more patients above the age of 16 and provide extractable data. Case reports, case series, systematic reviews, and meta-analyses were excluded.

### 2.3. Quality and Publication Bias Evaluation

The quality of the included studies was assessed using the Newcastle-Ottawa Quality Assessment Form for Cohort Studies. Each study could earn a maximum of 9 points and a minimum of 0 points. The points were divided into three categories: selection (4 points), comparability (2 points), and outcome (3 points). Two authors (O.G.C. and K.K.) performed quality assessments separately. Conflicts at this stage were resolved via arbitration by two more experienced authors (J.F. and T.S.). The publication bias evaluation was conducted by visual inspection of funnel plot asymmetry.

### 2.4. Statistical Analysis

Statistical analyses were performed using Review Manager 5.4.1 (The Cochrane Collaboration). Data about postoperative complications were extracted from included studies. These data were then grouped according to the type of complication (e.g., overall complications, infection, hydrocephalus, etc.). Certain studies did not report overall complications, in such cases, the sum of individual complications was used for analysis. The data were additionally categorized into “early” and “late” groups, according to the DC and CP interval provided in each study. A large portion of the literature, including previous meta-analyses, indicates the early CP time cutoff to be 90 days [4,5]. Some studies brought forth data about cranioplasties that would be considered “ultra-early”; thus, the authors performed subgroup analyses of studies in which CP was performed within 6 weeks after DC [6,7,8,9,10].

Dichotomized variables were pooled into an overriding odds ratio (OR) with a confidence interval (CI) of 95% using the Mantel–Haenszel method. The I^2^ statistic was used to calculate the data’s heterogeneity. Fixed-effects models were utilized if the data were found to be homogeneous; otherwise, random-effects models were applied. A *p*-value of less than 0.05 was considered statistically significant.

## 3. Results

### 3.1. Included Studies

**Figure 1:**
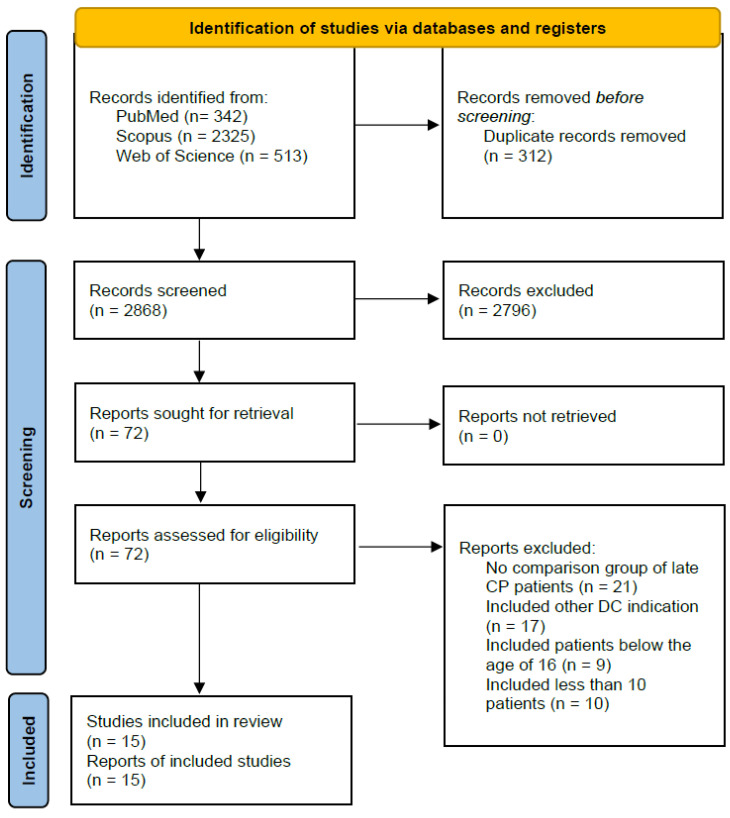
PRISMA flowchart.

After duplicate removal, 2868 studies were identified. Those studies were subjected to the first screening stage, prompting the removal of 2796 records. Studies that provided inestimable data were not included in the analyses. A total of 57 studies were excluded after the full-text screening; of these, 21 did not include a comparison group of late CP, 17 included other DC indications, 9 included patients below the age of 16, and 10 included less than 10 patients. If a study included mixed DC indications, but data concerning TBI patients were extractable, only their data were included in the review. Two studies reporting mixed DC indications did not provide explicit TBI patient data but were included in the review because the TBI data were included in previous reviews [4,5,11,12]. The patient demographic data, such as age and sex, were comparable within each study. Trauma severity was not mentioned across each study; thus, it could not be compared. The systematic review results are presented in the PRISMA flowchart (Figure 1).

### 3.2. Publication Bias

The funnel plot showed a slight asymmetry in the distribution of the included studies between the MD and the standard error (MD), suggesting potential publication bias (Supplementary Figure S1).

### 3.3. Overall Complications

**Figure 2:**
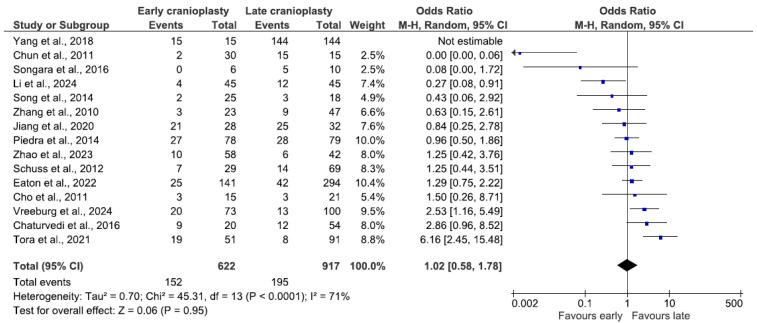
Forest plot of overall complications. Results indicate no difference in the odds of overall complications between groups.

Data from 14 studies were analyzed for overall complications, combining 1539 patients. Overall complications included all events mentioned in Table 1. The results of one study could not be included in this analysis, as the OR was not estimable due to complications in all patients [7]. The prevalence of complications in the early group reached 24.4% (n = 152/622) and 21.3% (n = 195/917) in the late group, with a combined prevalence of complications of 22.5% (n = 347/1539). There was no significant difference in the odds of complications between the early cranioplasty group and the late cranioplasty group (OR 1.02, 95% CI 0.58–1.78, *p* = 0.95; Figure 2). A random-effects model was used, as the data were heterogeneous (I^2^ = 71%, *p* < 0.0001).

**Figure 3:**
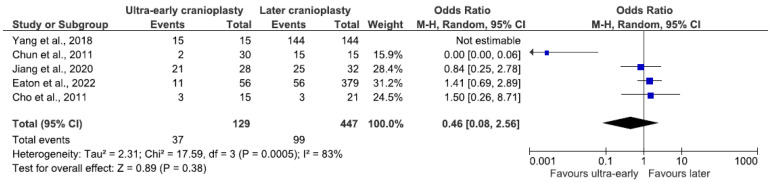
Forest plot of overall complications in the ultra-early subgroup. Results indicate no difference in the odds of overall complications between groups.

Subgroup analysis of the ultra-early group, containing 576 patients from five studies [6,7,8,9,10], revealed no statistically significant difference in overall complications between patients who underwent cranioplasty within 6 weeks of DC and those who underwent CP at a later time point (OR 0.46, 95% CI 0.08–2.56, *p* = 0.38; Figure 3), whilst trivially favoring ultra-early CP. The odds of complications in the ultra-early group totaled 28.7% (n = 37/129) while reaching only 22.1% (n = 99/447) in the later group. The combined odds of complications were 23.6% (n = 136/576). Due to significant data heterogeneity, a random-effects model was used (I^2^ = 83%, *p* = 0.0005).

### 3.4. Infection

Ten studies presented infectious complications, but only seven provided data that could be analyzed. A total of 1088 patient outcomes were included. The incidence of infections in the early group reached 8% (n = 31/388) and 8% (n = 56/700) in the late group. The difference in odds of pathogenic microbial complications between the early and late groups presented no statistical significance (OR 1.03, 95% CI 0.64–1.66, *p* = 0.9; Supplementary Figure S2) using a fixed-effects model, because of low heterogeneity (I^2^ = 29%, *p* = 0.21).

**Figure 4:**
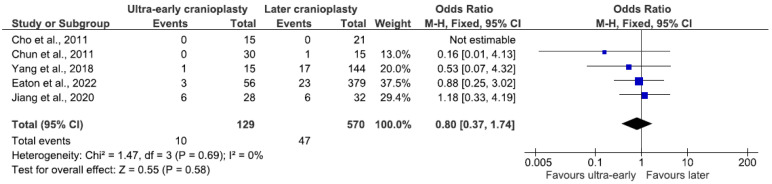
Forest plot of infections in the ultra-early subgroup. Results indicate no difference in the odds of infectious complications between groups.

Analysis of infections included in the ultra-early versus later groups, combining 699 patients from five studies, rendered an insignificant difference in odds, 7.8% (n = 10/129) and 8.2% (n = 47/570), respectively. No statistically significant difference was found between the two groups (OR 0.8, 95% CI 0.37–1.74, *p* = 0.58; Figure 4) using a fixed-effects model since data were homogenous (I^2^ = 0%, *p* = 0.69).

### 3.5. Hematoma

Five studies provided data about postoperative hematoma. Outcomes of 842 patients proved no difference in the odds of occurrence of such complications (OR 1.03, 95% CI 0.54–1.96, *p* = 0.93; Supplementary Figure S3). Due to data homogeneity, a fixed-effects model was used (I^2^ = 0%, *p* = 0.81). The incidence of hematoma was 5.4% (n = 19/350) in the early group and 4.7% (n = 23/492) in the late group.

Subgroup analysis of the ultra-early group provided a similar outcome (OR 1.07, 95% CI 0.5–2.29, *p* = 0.86; Supplementary Figure S4) using a fixed-effects model (I^2^ = 0%, *p* = 0.68). Hematoma was present in 11.1% (n = 11/99) of the ultra-early group and 8.6% (n = 48/555) in the later group. Data of 654 patients were analyzed.

### 3.6. Hydrocephalus

**Figure 5:**
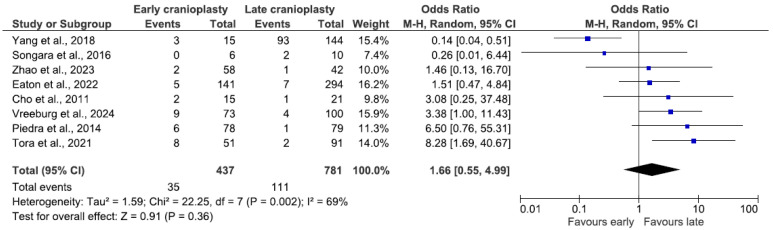
Forest plot of hydrocephalus. Results indicate no difference in the odds of hydrocephalus between groups.

Eight studies included hydrocephalus as a complication, combining data from 1218 patients. The combined occurrence of hydrocephalus was nearly 12% (n = 146/1218), 8% (n = 35/437) in the early group, and 14.2% (n = 111/781) in the late group. There was no statistically significant difference in the odds of developing hydrocephalus (OR 1.66, 95% CI 0.55–4.99, *p* = 0.36; Figure 5). A random-effects model was utilized, as the data showed significant signs of heterogeneity (I^2^ = 69%, *p* = 0.002).

**Figure 6:**
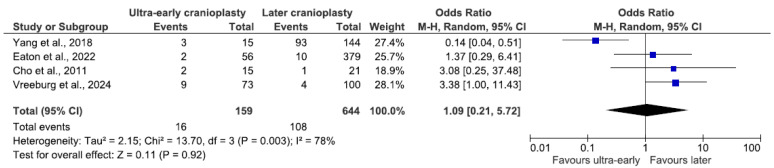
Forest plot of hydrocephalus in the ultra-early subgroup. Results indicate no difference in the odds of hydrocephalus between groups.

The analysis of hydrocephalus in the ultra-early group included 803 patients from four studies, proving no significant difference in the odds between the two groups (OR 1.09, 95% CI 0.21–5.72, *p* = 0.92; Figure 6). Due to significant data heterogeneity, a random-effects model was used (I^2^ = 78%, *p* = 0.003). The incidence of hydrocephalus reached 10% (n = 16/159) in the ultra-early group and 16.8% (n = 108/644) in the later group.

### 3.7. Seizure

Five studies provided data about seizure incidence in 794 patients. Using a fixed-effects model (I^2^ = 0%, *p* = 0.62), no significant difference in odds was found between the early and late groups (OR 1.04, 95% CI 0.57–1.92, *p* = 0.89; Supplementary Figure S5). The combined occurrence of seizures was 5.9% (n = 47/794).

Subgroup analysis of the ultra-early group, including 608 patients from two studies, indicated no significant difference in the odds of seizures between the groups (OR 1.56, 95% CI 0.75–3.28, *p* = 0.24; Supplementary Figure S6), while trivially favoring the later group. A fixed-effects model was used due to data homogeneity (I^2^ = 0%, *p* = 0.56). The combined occurrence of seizures was 6.6% (n = 40/608).

### 3.8. Wound Healing Disturbance

Five studies provided data about wound healing disturbance in 462 patients. The incidence of such complications was 5% (n = 23/462). There was no significant difference in the odds of wound healing disturbance between the early and late CP groups (OR 0.9, 95% CI 0.37–2.17, *p* = 0.81; Supplementary Figure S7). This complication category was not subjected to subgroup analysis due to insufficient data in the ultra-early CP group.

### 3.9. Hygroma

**Figure 7:**
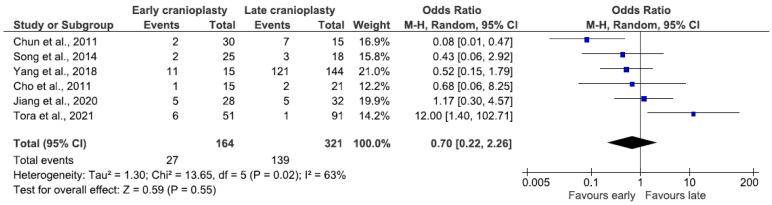
Forest plot of hygroma. Results indicate no difference in the odds of hygroma between groups.

Six studies reported outcomes of 485 patients, resulting in a combined incidence of hygroma of 34.2% (n = 166/485). There was no significant difference in the odds of hygroma between the early and late CP groups (OR 0.7, 95% CI 0.22–2.26, *p* = 0.55; Figure 7).

Subgroup analysis of the ultra-early group included 300 patients and insignificantly favored the ultra-early group (OR 0.45, 95% CI 0.15–1.37, *p* = 0.16; Supplementary Figure S8). The combined prevalence of hygroma in this subgroup was 51.3% (n = 154/300), 21.6% (n = 19/88) in the ultra-early group, and 63.7% (n = 135/212) in the later group.

## 4. Discussion

### 4.1. Overall Complications

Our study’s combined odds of complications are 22.5%, which is comparable to the findings of Zheng et al. [4] (24,9%) and Malcolm et al. [5] (25,7% in the trauma group). The review of Yadla et al. [20] included only two studies with mixed indications for DC, but reported similar results (20%). Different indications for DC may slightly affect the odds of overall complications [21]. In contrast, a review performed by Kurland et al. [22] of the pediatric population presented substantially lower overall complication rates (6.4%). This may be attributed to fewer comorbidities [23] and simpler surgical context due to smaller defect sizes [24]. Subgroup analysis showed insignificant favoring of the ultra-early group. These results may be attributed to the findings from the study of Chun et al. [9], who examined CP within a month of DC. The credibility of this parameter could be increased if future studies clearly stated the overall complications, as most of the studies included in this review did not.

### 4.2. Infection

The pooled infection rate in our study is 8%, and 8.2% in the ultra-early subgroup. These findings are comparable to those of other reviews. Malcolm et al. [5] and Zheng et al. [4] reported slightly different rates (7.7% in the trauma group and 8.9%, respectively). The prevalence of infections in CP patients may depend on how autologous bone grafts are stored and the use of preventive antibiotic treatment. Subcutaneous preservation of cryopreservation of autologous bone grafts does not affect the infection rates in trauma CP patients [25]. Abode-Iyamah et al. [26] studied the effects of vancomycin powder on postoperative infections in CP patients with mixed indications and presented no significant differences from the control group, subjected to standard prophylaxis using nafcillin or cefazolin. Further studies examining infection rate reduction techniques in trauma CP patients could provide additional data to improve postoperative outcomes.

### 4.3. Hematoma

In this review, we report a 5% rate of hematoma. This finding is comparable to the previous review by Kurland et al. [22] in the pediatric population (3.6%). Malcolm et al. [5] examined intracranial hemorrhage in trauma CP patients but only included one study [2], which was also included in our review. Interestingly, hematoma rates in the ultra-early subgroup analysis varied between the ultra-early and later groups (a difference of 2.5%) but with no statistical significance.

### 4.4. Hydrocephalus

Our study’s combined odds of hydrocephalus are 12%, significantly higher than Malcolm et al.’s [5] findings (5% in the trauma group), which included only two studies that are also included in our review [2,6]. According to Kurland et al.’s [22] review, the odds of cerebrospinal fluid disturbances were also lower in the pediatric population (5.4%). Analysis of the ultra-early subgroup resulted in a lower incidence rate in the ultra-early group. Previous reviews report lower occurrences of hydrocephalus with earlier CP [27,28].

### 4.5. Seizure

This review presents the odds of postoperative seizures in trauma CP patients as 5.9%. Subgroup analysis of the ultra-early group revealed the odds to be slightly higher, at 6.6%. These findings seem aligned with those of Yao et al. [29], who estimated a higher rate of seizures with earlier CP. Data about seizure incidence should be approached carefully, as it is known that retrospective studies report substantially lower seizure rates than prospective studies [29]. Interestingly, there is no difference in new-onset post-CP seizures between patients with different DC indications [21].

### 4.6. Wound Healing Disturbance

Our study’s combined odds of wound healing disturbance was 5%. No subgroup analysis was performed for this type of complication because only one study in the ultra-early group accounted for wound healing disturbance, and there was no significant difference between groups with an ultra-early CP time cutoff of 6 weeks [10]. Several definitions were grouped under the wound healing disturbance category: wound healing disturbance [18,19], scalp necrosis [10], subcutaneous necrosis [13], and dehiscence [16]. To our knowledge, no other review analyzed wound healing disturbance as a complication of CP. We speculate wound healing disturbance rates are higher in TBI patients than in non-TBI patients due to potential breaks in skin continuity, which may introduce infectious microorganisms and increase wound dehiscence [30].

### 4.7. Hygroma

The pooled rate of hygroma in our study was 34.2%. Subgroup analysis revealed interesting discrepancies between the ultra-early group (21.6%) and later group (63.7%). It is important to note that the study of Yang et al. [7] counted contralateral hematoma and hygroma together while counting ipsilateral or interhemispheric hygroma as a separate data point. This difference is not statistically significant, but future studies may help to clarify these data.

### 4.8. Limitations

This meta-analysis presents several limitations. The reporting of complications varies between centers and may result from various factors. Certain studies did not focus on reporting overall complications, forcing the authors to sum individually listed complications, which may create inaccuracies in data. The definitions of complications such as infection and hygroma were also heterogeneous, prompting the aggregation of complications in general categories, whether the complication required reoperation or was managed non-surgically.

The included studies reported diverse DC and CP techniques. The anatomical location of surgeries differed between studies, and various extensions of those surgeries were presented, which plays a role in patients’ outcomes. A strength of this meta-analysis is the inclusion of studies that presented solely traumatic indications for DC, providing new evidence on the efficiency of CP in such cases.

Timing of CP varied between the included studies, with most of them considering early CP to be performed within 90 days. The dichotomization of CP timing into early and late categories provides a good overall suggestion about the optimal timing of CP but does not result in a definitive answer. Subgroup analysis of the ultra-early cohort was possible because several studies provided data about CP being performed within 6 weeks of DC.

This review included one study with a poor quality rating on the Newcastle-Ottawa Scale. The other fourteen studies were rated as good or moderate quality (Supplementary Table S1).

## 5. Conclusions

This systematic review and meta-analysis, evaluating postoperative complications after CP in patients who sustained TBI, is the most extensive available analysis of this subject. The results suggest no significant difference in the odds of complications between early and late CP, and early CP can be beneficial for patients at risk of developing hydrocephalus. Ultra-early CP may lower the odds of overall complications, especially hygroma, but is a more significant risk factor for postoperative seizures. Although these results should be addressed with caution, they may be of value to physicians deciding on CP timing.

## Figures and Tables

**Table 1:** Characteristics of included studies.

Authors & Year	Country	DC Indication	Early CP Cutoff	Number of Patients			Reported Complications
				Early	Late	Total	
Chaturvedi et al., 2016 [12]	India	TBI	90	20	54	74	N/A
Cho et al., 2011 [6]	South Korea	TBI	6 weeks	15	21	36	Infection, subdural fluid collection, ventriculomegaly
Chun et al., 2011 [9]	South Korea	TBI	30	30	15	45	Infection, Subdural fluid collection, dura tear, soft tissue injury, inadequate dissection
Eaton et al., 2022 [8]	USA	TBI	90	141	294 (I + L)	435	Hydrocephalus, seizure, hematoma, infection
Jiang et al., 2020 [10]	China	TBI	6 weeks	28	32	60	Infection, fluid accumulation, scalp necrosis, hematoma
Li et al., 2024 [13]	China	TBI	90	45	45	90	Infection, hemorrhage, subcutaneous necrosis, subcutaneous effusion, chewing discomfort
Piedra et al., 2014 [2]	USA	TBI	90	78	79	157	Hydrocephalus, infection, hematoma, bone graft resorption
Schuss et al., 2012 [11]	Germany	TBI	2 months	29	69	98	EDH or SDH, Wound d healing disturbance, Abscess, Hygroma, CSF fistula, Other
Song et al., 2014 [14]	China	TBI	12 weeks	25	18	43	Subdural fluid collection
Songara et al., 2016 [15]	India	TBI	90	6	10	16	Hydrocephalus, seizure, trauma to operative site
Tora et al., 2021 [16]	USA	TBI	90	51	91	142	Infection, wound dehiscence, reoperation, hydrocephalus, ischemic stroke, resorption, extra-axial (fluid) collection
Vreeburg et al., 2024 [17]	Netherlands	TBI	90	73	100	173	Hydrocephalus, seizure
Yang et al., 2018 [7]	South Korea	TBI	35	15	144	159	Sinking skin flap syndrome, ventriculomegaly, shunt-dependent hydrocephalus, Ipsilateral or interhemispheric hygroma, Contralateral hematoma or hygroma, Revision due to the infection
Zhang et al., 2010 [18]	China	TBI	90	23	47	70	Fluid below skin flap, infection, wound healing disturbance, seizure
Zhao et al., 2023 [19]	China	TBI	6 months	58	42	100	EDH or SDH, wound healing complications, hydrocephalus, seizure

EDH = epidural hematoma; SDH = subdural hematoma; CSF = cerebrospinal fluid.

## Supplementary Materials

The following supporting information can be downloaded at: https://www.mdpi.com/article/10.3390/jcm14124176/s1, Figure S1: Funnel plot of included studies suggesting potential publication bias. Figure S2: Forest plot of infections. Results indicate no difference in the odds of infectious complications between groups. Figure S3: Forest plot of hematoma. Results indicate no difference in the odds of hematoma between groups. Figure S4: Forest plot of hematoma in the ultra-early subgroup. Results indicate no difference in the odds of hematoma between groups. Figure S5: Forest plot of seizure. Results indicate no difference in the odds of seizure between groups. Figure S6: Forest plot of seizure in the ultra-early subgroup. Results indicate no difference in the odds of seizure between groups. Figure S7: Forest plot of wound healing disturbance in the ultra-early subgroup. Results indicate no difference in the odds of wound healing disturbance between groups. Figure S8: Forest plot of hydroma in the ultra-early subgroup. Results indicate no difference in the odds of hydroma between groups. Table S1: Included studies and their quality rating using the Newcastle-Ottawa Scale.
