# Supplementary material for: Comparison of Complications in Early and Late Cranioplasty Following Decompressive Craniectomy Due to Traumatic Brain Injury: Systematic Review and Meta-Analysis
Source: J Clin Med. 2025 Jun 12;14(12):4176. doi: 10.3390/jcm14124176 (PMC12194293; doi:10.3390/jcm14124176)
Supplement: Supplementary file 1 [file jcm-14-04176-s001.zip › Supplementary Table S1.pdf]

| Reference | Selection | | | | Comparability | Outcome | | | Results | |
|---|---|---|---|---|---|---|---|---|---|---|
| Study | Representativeness of the exposed cohort | Selection of the non-exposed cohort | Ascertainment of exposure | Demonstration that outcome of interest was not present at start of study | Comparability of cohorts on the basis of the design or analysis controlled for confounders | Assessment of outcome | Was follow-up long enough for outcomes to occur | Adequacy of follow-up of cohorts | Total score | Rating |
| Chaturvedi et al., 2016[12] | + | - | + | + | -+ | + | + | + | 7/9 | Good quality |
| Cho et al., 2011[6] | + | + | + | + | -- | + | + | + | 7/9 | Moderate quality |
| Chun et al., 2011[9] | + | + | + | + | -- | + | + | - | 6/9 | Moderate quality |
| Eaton et al., 2022[8] | + | + | + | + | ++ | + | + | - | 8/9 | Good quality |
| Jiang et al., 2020[10] | + | + | + | + | -+ | + | + | - | 7/9 | Good quality |
| Li et al., 2024[13] | + | + | + | + | -+ | + | + | - | 7/9 | Good quality |
| Piedra et al., 2014[2] | + | + | + | + | ++ | + | + | + | 9/9 | Good quality |
| Schuss et al., 2012[11] | + | + | + | + | ++ | + | - | - | 7/9 | Good quality |
| Song et al., 2014[14] | + | + | + | + | -+ | + | + | + | 8/9 | Good quality |
| Songara et al., 2016[15] | - | - | + | - | -- | + | - | - | 2/9 | Poor quality |
| Tora et al., 2021[16] | + | + | + | - | -+ | + | + | + | 8/9 | Good quality |
| Vreeburg et al., 2024[17] | + | + | + | + | ++ | + | + | + | 9/9 | Good quality |
| Yang et al., 2018[7] | + | - | + | + | -+ | + | + | - | 6/9 | Moderate quality |
| Zhang et al., 2010[18] | + | + | + | + | -+ | + | + | + | 8/9 | Good quality |
| Zhao et al., 2023[19] | + | + | + | + | -+ | + | + | + | 8/9 | Good quality |

*Supplementary Table S1. Included studies and their quality rating using the Newcastle-Ottawa Scale.*
