# Supplementary figures and images for: Comparison of Complications in Early and Late Cranioplasty Following Decompressive Craniectomy Due to Traumatic Brain Injury: Systematic Review and Meta-Analysis
Source: J Clin Med. 2025 Jun 12;14(12):4176. doi: 10.3390/jcm14124176 (PMC12194293; doi:10.3390/jcm14124176)

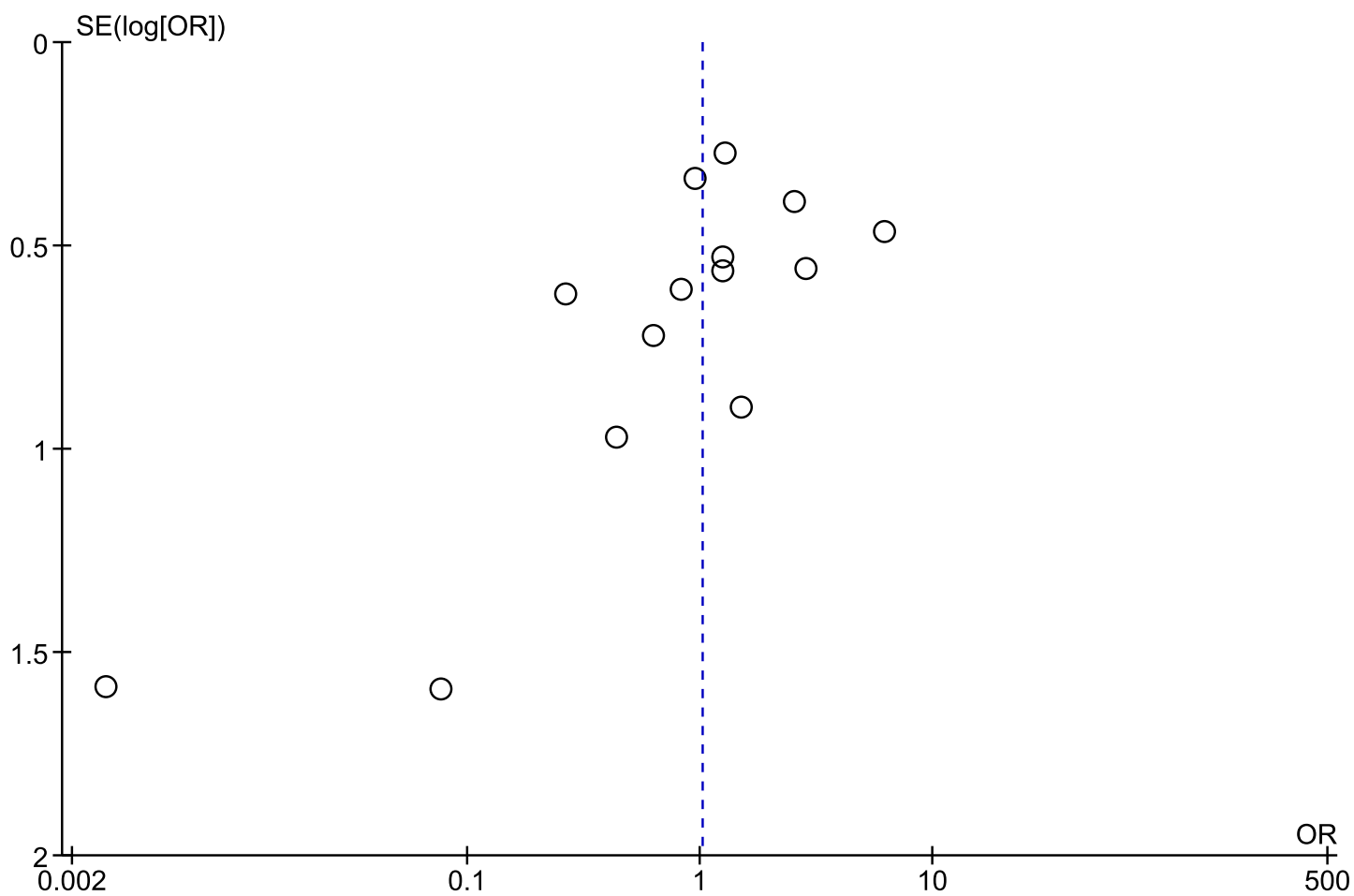

Supplement: Supplementary file 1 [file jcm-14-04176-s001.zip › Supplementary Figure S1.pdf]

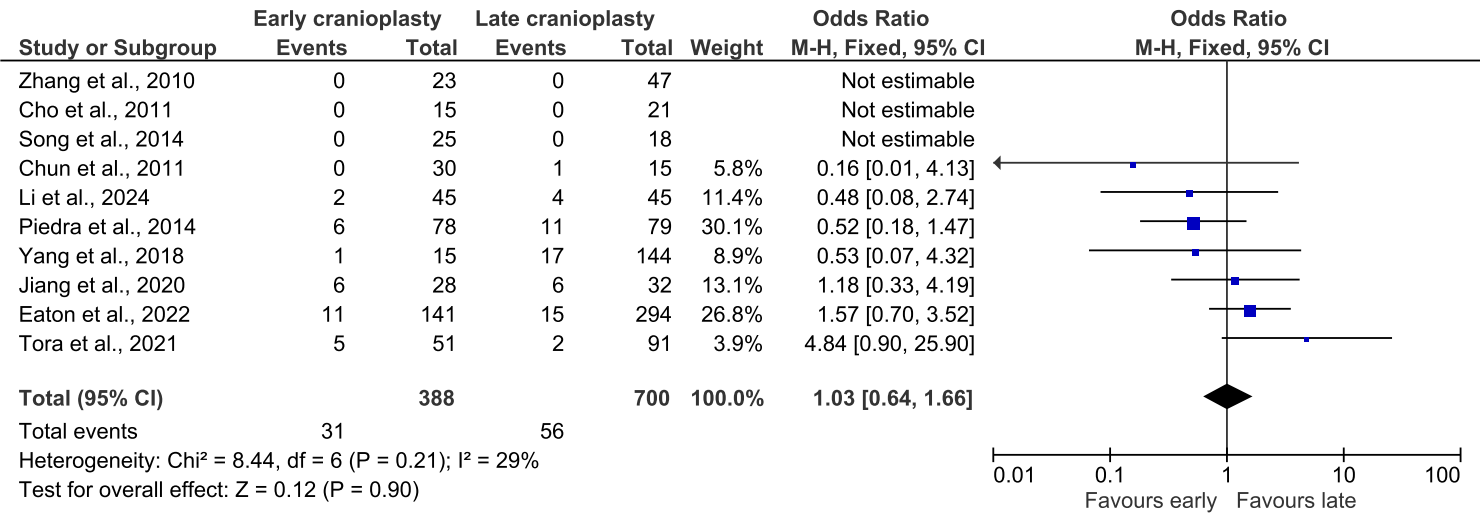

Supplement: Supplementary file 1 [file jcm-14-04176-s001.zip › Supplementary Figure S2.pdf]

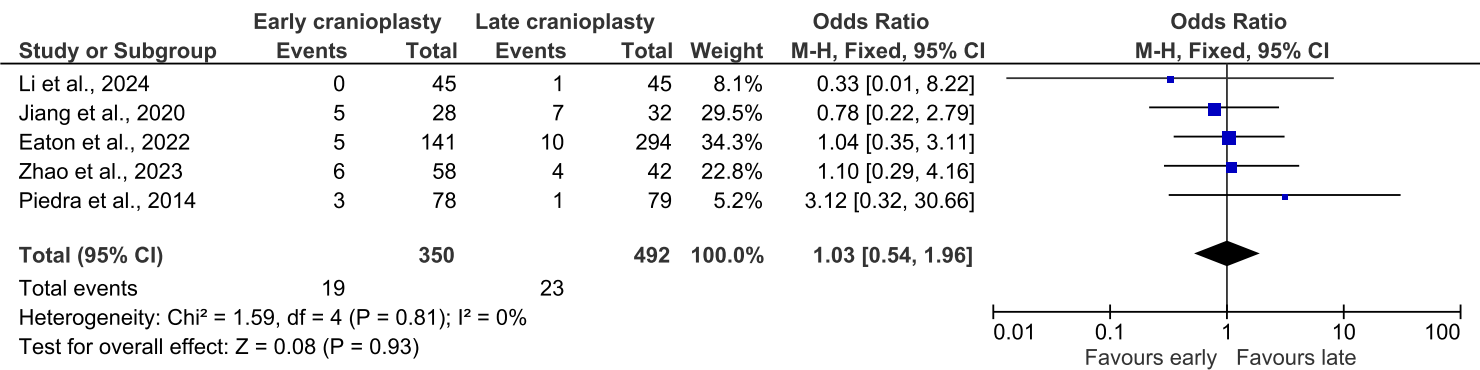

Supplement: Supplementary file 1 [file jcm-14-04176-s001.zip › Supplementary Figure S3.pdf]

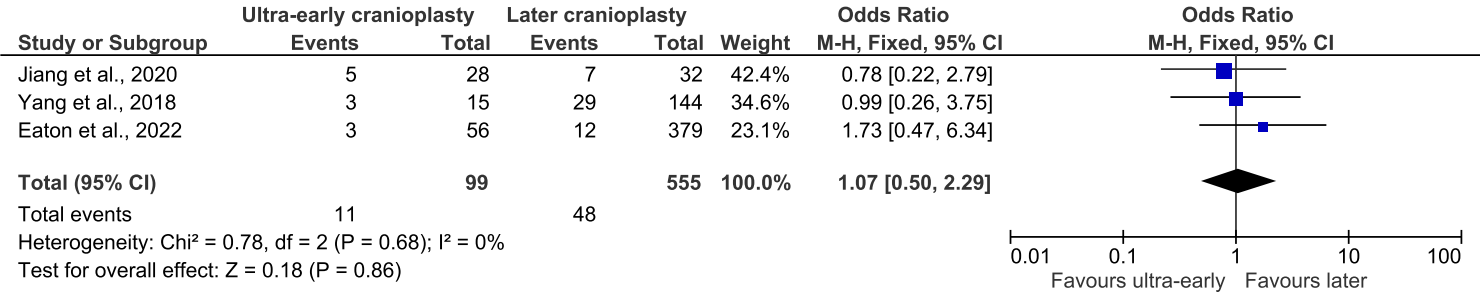

Supplement: Supplementary file 1 [file jcm-14-04176-s001.zip › Supplementary Figure S4.pdf]

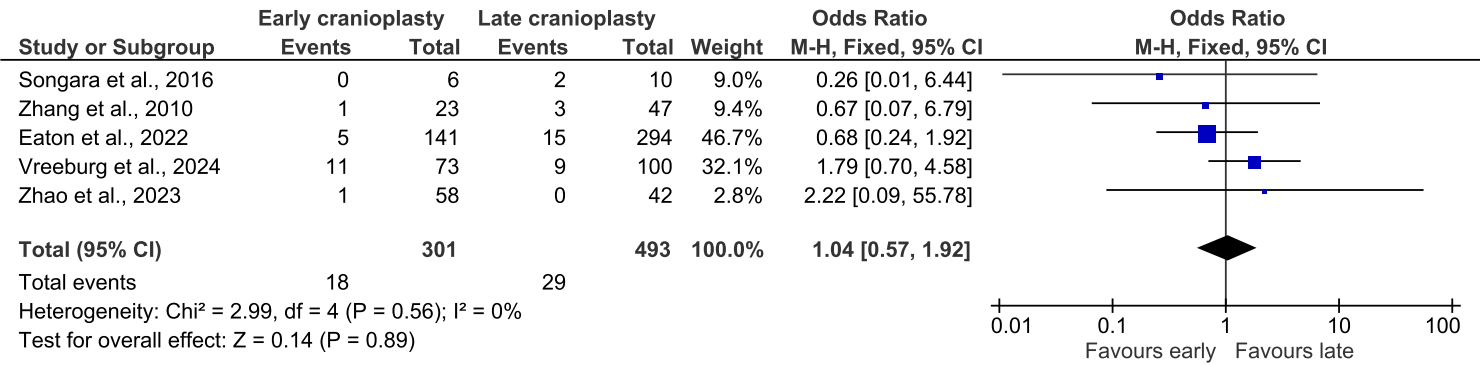

Supplement: Supplementary file 1 [file jcm-14-04176-s001.zip › Supplementary Figure S5.pdf]

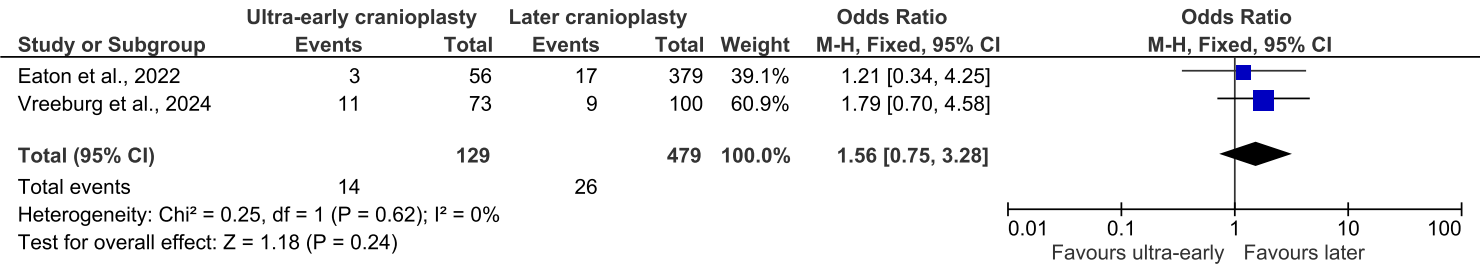

Supplement: Supplementary file 1 [file jcm-14-04176-s001.zip › Supplementary Figure S6.pdf]

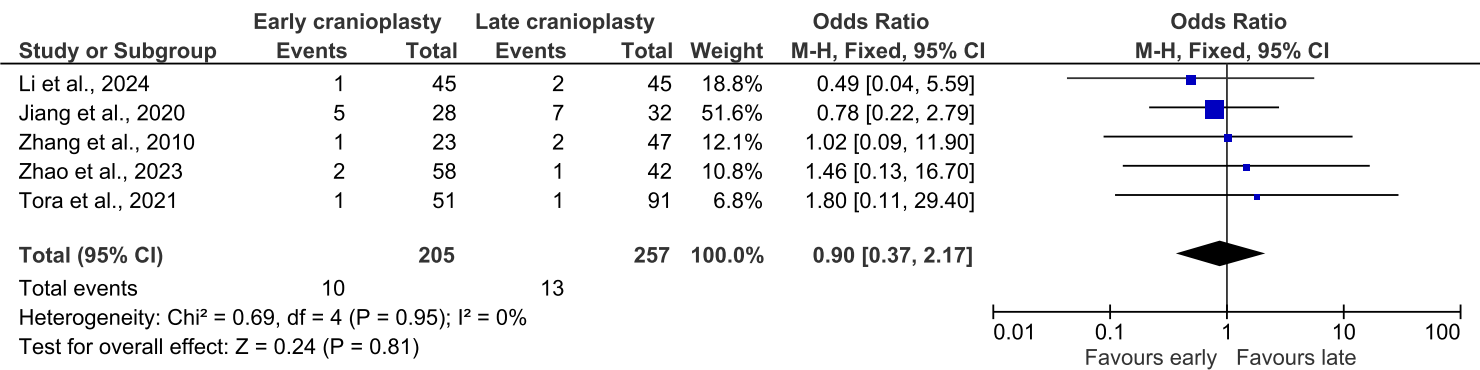

Supplement: Supplementary file 1 [file jcm-14-04176-s001.zip › Supplementary Figure S7.pdf]

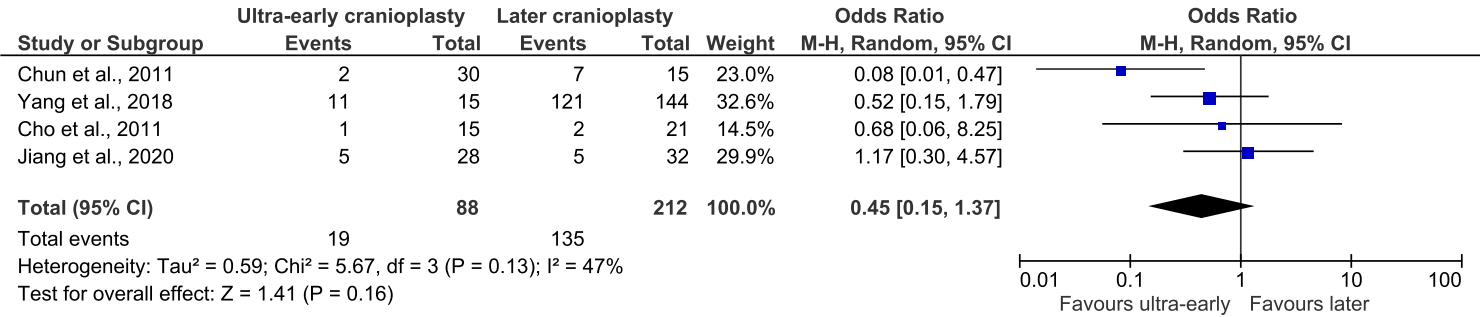

Supplement: Supplementary file 1 [file jcm-14-04176-s001.zip › Supplementary Figure S8.pdf]
